# Clinicopathological Features of Stage I–III Colorectal Cancer Recurrence Over 5 Years After Radical Surgery Without Receiving Neoadjuvant Therapy: Evidence From a Large Sample Study

**DOI:** 10.3389/fsurg.2021.666400

**Published:** 2021-08-09

**Authors:** Dakui Luo, Yufei Yang, Zezhi Shan, Qi Liu, Sanjun Cai, Qingguo Li, Xinxiang Li

**Affiliations:** ^1^Department of Colorectal Surgery, Fudan University Shanghai Cancer Center, Shanghai, China; ^2^Department of Oncology, Shanghai Medical College, Fudan University, Shanghai, China

**Keywords:** late recurrence, colorectal cancer, radical surgery, early recurrence, clinicopathological features

## Abstract

Late recurrence (5 or more years) after radical resection of colorectal cancer (CRC) is rare. This study aims to investigate the features of late recurrence in stage I–III CRC. A total of 9,754 stage I–III patients with CRC who underwent radical surgery without receiving neoadjuvant therapy, at the Fudan University Shanghai Cancer Center (FUSCC), were enrolled in this study. These patients were divided into three groups: early recurrence (3 months−2 years), intermediate recurrence (2–5 years), and late recurrence (over 5 years). The median duration of follow-up was 53.5 ± 30.1 months. A total of 2,341 (24.0%) patients developed recurrence. The late recurrence rate was 11.7%. Patients with a higher risk of late recurrence were more likely to be older, to be at the T4 stage, to have a higher degree of colon cancer, to have a lower frequency of signet ring cell carcinoma, to have fewer poorly differentiated tumors, to be at the early stage of CRC, along with less perineural and vascular invasions. Multivariate logistic regression analysis identified age, differentiation, T stage, N stage, perineural, and vascular invasions as independent factors for late recurrence. Late recurrent CRC has some distinctive characteristics. Although recurrence over 5 years after surgery is infrequent, an enhanced follow-up is still needed for the selected patients after 5 years.

## Introduction

Colorectal cancer (CRC) is the third most common malignancy and the second most common cause of cancer-related mortality worldwide (1). For resectable non-metastatic CRC, surgery with bowel resection and removal of the regional lymph nodes is preferred. Adjuvant therapy is administrated according to the postoperative pathological stage. Posttreatment surveillance is regularly performed to identify a recurrence that is potentially resectable for the cure. Although receiving standard treatment, about 25–40% of patients still suffer tumor recurrence during follow-up due to high spatiotemporal heterogeneity (2–4). The risk of relapse largely depends on the tumor, node, metastases (TNM) stage, and several other important clinicopathological factors (5). Previous evidence indicated that 80% of recurrences occurred in the first 3 years and 95% of them occurred in the first 5 years after curative surgery (6–8). In general, early recurrence was defined as recurrence within 2 years of surgery. Early recurrence is majorly ascribed to adverse clinicopathological characteristics and resistance to adjuvant chemotherapy. Most surveillances compromise over 5 years after curative surgery. However, some relapses were detected after 5 years. It is necessary to identify the characteristics of recurrent CRC that occurred >5 years and to implement enhanced follow-up programs.

## Materials and Methods

### Study Population

During the months between January 2008 and May 2018, a total of 13,765 patients with CRC were identified from the Fudan University Shanghai Cancer Center (FUSCC) database. In this study, the inclusion criteria were as follows: (1) patients had stage I–III diseases, patients in the T stage or the undetermined TNM stage were excluded; (2) patients had undergone curative surgery; (3) patients did not undergo neoadjuvant therapy; (4) the histology presented with adenocarcinoma, mucinous adenocarcinoma, or signet ring cell carcinoma; (5) survival information was available; and (6) the disease-free, survival period was longer than 3 months. A total of 4,011 patients were excluded due to their unknown pathological stage, receiving salvage surgery for local excision without the evidence of tumor cells, receiving neoadjuvant therapy, or without active follow-up. The following clinicopathological characteristics were extracted from the FUSCC database: age at diagnosis, gender, tumor location, histologic type, histological differentiation, T stage, N stage, pathological stage (AJCC 8th Edition), perineural invasion, vascular invasion, and survival information. This study was approved by the Ethics Committee and Institutional Review Board of the FUSCC and written informed consent was obtained from all the patients.

### Treatment and Follow-up

All patients underwent standard curative surgery in accordance with the clinical guidelines. Total mesorectal excision or complete mesocolic excision was performed. Adjuvant therapy was adopted in selected patients with stage II and in all stage III patients who were capable of tolerating the treatment. In general, patients with low-risk stage II disease can be considered for adjuvant therapy with capecitabine alone or with observation, while patients with high-risk stage II and stage III disease can be considered for adjuvant chemotherapy with CapeOX (oxaliplatin and capecitabine). Recurrence includes local recurrence and distant metastasis. Clinical or radiological detection was accepted and histopathological confirmation was not mandatory. The diagnosis should be evaluated by the multidisciplinary team. Review of the medical records, follow-ups *via* telephone, and data linkage of the death registry were employed for collecting the survival data. The last follow-up date was November 30, 2019.

### Statistical Analysis

The patients were divided into three periods of recurrences, namely early recurrence (3 months−2 years), intermediate recurrence (2–5 years), and late recurrence (over 5 years). The categorical variables were analyzed by the chi-squared test. Univariate and multivariate ordinal logistic regression models or multinomial logistic regression models were used to evaluate the potential factors associated with the recurrence time. The patients were also divided into two groups (<2 years, >2 years or <5 years, > 5 years), and univariate and multivariate binary logistic regression models were adopted to identify the potential factors associated with the recurrence time. The Kaplan–Meier method was utilized to plot the survival curves, and the survival difference was determined using the log-rank test. All statistical analyses were performed with SPSS 25.0.

## Results

### Characteristics and Survival in Early Recurrence, Intermediate Recurrence, and Late Recurrence

**Figure 1:**
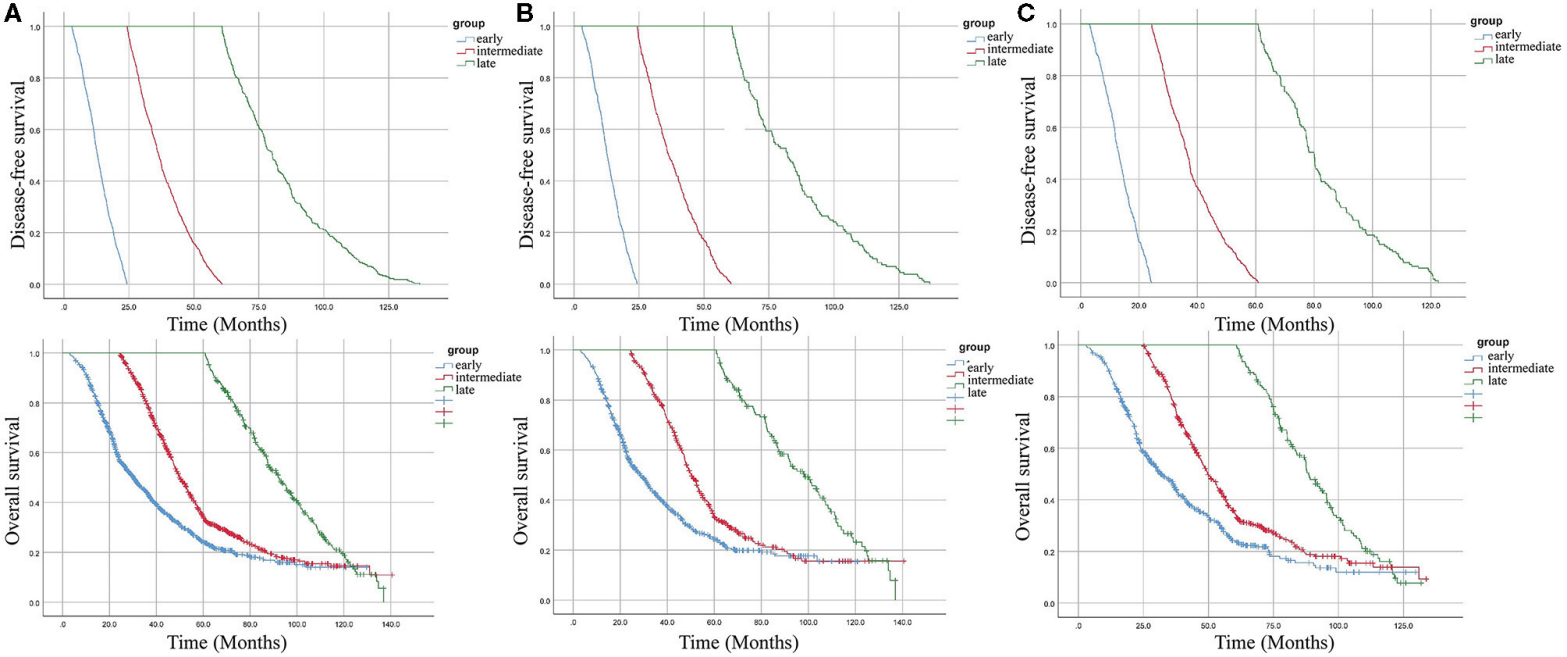
Kaplan–Meier curves for disease-free survival (DFS) and overall survival (OS) according to the time taken for recurrence. **(A)** overall cohort. **(B)** patients with colon cancer. **(C)** patients with rectal cancer.

A total of 9,754 eligible patients were identified in the study. The median duration of follow-up was 53.5 ± 30.1 months. During the surveillance, 2,341 patients experienced recurrence. These patients were divided into three groups: early recurrence (3 months−2 years, *N* = 1,187), intermediate recurrence (2–5 years, *N* = 849), and late recurrence (over 5 years, *N* = 274), after initial surgery. In this study, the overall recurrence rate after curative surgery was 24.0% (2341/9754) in stage I–III CRC without neoadjuvant therapy. The early recurrence rate was 50.7% (1187/2341) and the late recurrence rate was 11.7% (274/2341). The clinicopathological features in the different groups are shown in Table 1. Clinicopathological features of different time intervals to recurrence were shown in Supplementary Tables 1, 2 according to tumor location. Kaplan–Meier curves were plotted based on three groups (Figure 1). As expected, a significantly increased 5-year overall survival (OS) rate was observed with prolonged recurrence time (colon cancer: 24.4, 33.2, 100%, *p* < 0.001; rectal cancer: 23.8, 34.8, 100%, *p* < 0.001; overall cohort: 24.2, 34.3, 100%, *p* < 0.001).

**Table 1:** Clinicopathological features of stage I–III colorectal cancer (CRC) recurrence according to postoperative time.

**Variables**		**Recurrence**	
	<2 years (*N* = 1187)	2–5 years (*N* = 849)	>5 years (*N* = 274)
**Age**			
≤ 60	544 (45.8%)	326 (38.4%)	83 (30.6%)
>60	643 (54.2%)	523 (61.6%)	188 (69.4%)
**Gender**			
Male	715 (60.2%)	514 (60.7%)	164 (59.9%)
Female	472 (39.8%)	334 (39.3%)	110 (40.1%)
**Location**			
Colon cancer	629 (53.0%)	390 (45.9%)	133 (48.5%)
Rectal cancer	558 (47.0%)	459 (54.1%)	141 (51.5%)
**Histologic type**			
Adenocarcinoma	983 (82.8%)	721 (84.9%)	230 (83.9%)
Mucinous	149 (12.6%)	112 (13.2%)	42 (15.3%)
Signet ring cell	55 (4.6%)	16 (1.9%)	2 (0.7%)
**Differentiation**			
Poor	411 (34.6%)	200 (23.6%)	47 (17.2%)
Moderate	730 (61.5%)	617 (72.7%)	210 (76.6%)
Well	6 (0.5%)	13 (1.5%)	5 (1.8%)
Unknown	40 (3.4%)	19 (2.2%)	12 (4.4%)
**T stage**			
T1	24 (2.0%)	27 (3.2%)	12 (4.4%)
T2	113 (9.5%)	143 (16.8%)	57 (20.8%)
T3	379 (31.9%)	143 (16.8%)	3 (1.1%)
T4	671 (56.5)	536 (63.1%)	202 (73.7%)
**N stage**			
N0	368 (31.0%)	368 (43.3%)	154 (56.2%)
N1	395 (33.3%)	297 (35.0%)	87 (31.8%)
N2	424 (35.7%)	184 (21.7%)	33 (12.0%)
**TNM stage**			
I	90 (7.6%)	125 (14.7%)	52 (19.0%)
II	278 (23.4%)	243 (28.6%)	102 (37.2%)
III	819 (69.0%)	481 (56.7%)	120 (43.8%)
**Perineural invasion**			
Negative	723 (60.9%)	620 (73.0%)	239 (87.1%)
Positive	464 (39.1%)	229 (27.0%)	35 (12.8%)
**Vascular invasion**			
Negative	659 (55.5%)	600 (70.7%)	227 (82.8%)
Positive	528 (44.5%)	249 (29.3%)	47 (17.2%)

### Recurrence Pattern of Patients With Late Recurrence

Most patients in the late recurrence group were over 60 years of age. Colon cancer, non-signet ring cell carcinoma, tumors with a well-differentiated histological type, lack of lymph node metastasis, stage I disease, and no evidence of perineural and vascular invasions were the more frequently demonstrated characteristics in the late recurrence group. Compared with patients with T4, patients with T2 had a higher risk of developing late recurrence, while patients with T3 had a lower risk. There was no difference between the three groups in terms of gender (Table 2). Multivariate logistic regression analysis identified age, differentiation, T stage, N stage, perineural and vascular invasions as independent factors for late recurrence (Table 3).

**Table 2:** Univariate ordinal logistic regression or multinomial logistic regression analysis of the factors associated with recurrences <2 years, 2–5 years, and >5 years after radical surgery (<2 years as a reference).

**Variables**	**Crude OR (95%CI)**	***P* value**
**Age**		
≤ 60	0.664 (0.566–0.781)	<0.001
>60	Reference	
**Gender**		
Male	1.003 (0.855–1.178)	0.967
Female	Reference	
**Location**		
Colon cancer	1.246 (1.065–1.458)	0.006
Rectal cancer	Reference	
**Histologic type**		
Adenocarcinoma	3.015 (1.758–5.170)	<0.001
Mucinous	3.297 (1.857–5.854)	<0.001
Signet ring cell	Reference	
**Differentiation**		
Poor	0.243 (0.113–0.521)	<0.001
Moderate	0.462 (0.218–0.981)	0.044
Well	Reference	
**T stage**		
**2–5 years**		
T1	1.408 (0.803–2.469)	0.232
T2	1.584 (1.207–2.079)	0.001
T3	0.472 (0.378–0.591)	<0.001
T4	Reference	
**>5 years**		
T1	1.661 (0.816–3.380)	0.162
T2	1.676 (1.175–2.390)	0.004
T3	0.026 (0.008–0.083)	<0.001
T4	Reference	
**N stage**		
N0	2.904 (2.365–3.566)	<0.001
N1	1.923 (1.557–2.375)	<0.001
N2	Reference	
**TNM stage**		
I	2.612 (2.038–3.349)	<0.001
II	1.777 (1.483–2.130)	<0.001
III	Reference	
**Perineural invasion**		
** <2 years**		
Negative	1.738 (1.435–2.104)	<0.001
Positive	Reference	
**2–5 years**		
Negative	4.382 (3.017–6.366)	<0.001
Positive	Reference	
**Vascular invasion**		
Negative	2.321 (1.955–2.754)	<0.001
Positive	Reference	

**Table 3:** Multivariate multinomial logistic regression analysis of the factors associated with recurrences <2 years, 2–5 years, and >5 years after radical surgery (>5 years as a reference).

**Variables**	**Adjusted OR (95%CI)**	***P* value**
**Age**		
** <2 years**		
≤ 60	1.804 (1.336–2.440)	<0.001
>60	Reference	
**2–5 years**		
≤ 60	1.354 (1.001–1.832)	0.049
>60	Reference	
**Location**		
** <2 years**		
Colon cancer	0.830 (0.621–1.108)	0.206
Rectal cancer	Reference	
**2–5 years**		
Colon cancer	1.084 (0.813–1.445)	0.582
Rectal cancer	Reference	
**Histologic type**		
** <2 years**		
Adenocarcinoma	0.323 (0.042–2.493)	0.279
Mucinous	0.221 (0.028–1.713)	0.148
Signet ring cell	Reference	
**2–5 years**		
Adenocarcinoma	0.374 (0.047–2.979)	0.353
Mucinous	0.347 (0.043–2.780)	0.319
Signet ring cell	Reference	
**Differentiation**		
** <2 years**		
Poor	4.737 (1.228–18.271)	0.024
Moderate	2.773 (0.753–10.211)	0.125
Well	Reference	
**2–5 years**		
Poor	1.411 (0.436–4.563)	0.566
Moderate	1.129 (0.369–3.456)	0.831
Well	Reference	
**T stage**		
** <2 years**		
T1	0.892 (0.337–2.356)	0.817
T2	0.692 (0.371–1.292)	0.248
T3	38.721 (12.237–122.527)	<0.001
T4	Reference	
**2**–**5 years**		
T1	0.777 (0.301–2.011)	0.604
T2	0.841 (0.454–1.557)	0.582
T3	17.864 (5.617–56.819)	<0.001
T4	Reference	
**N stage**		
** <2 years**		
N0	0.409 (0.250–0.667)	<0.001
N1	0.601 (0.380–0.951)	0.030
N2	Reference	
**2**–**5 years**		
N0	0.617 (0.376–1.014)	0.057
N1	0.819 (0.513–1.308)	0.404
N2	Reference	
**TNM stage**		
** <2 years**		
I	1.765 (0.830–3.751)	0.140
II	/	/
III	Reference	
**2**–**5 years**		
I	1.674 (0.802–3.492)	0.170
II	/	/
III	Reference	
**Perineural invasion**		
** <2 years**		
Negative	0.456 (0.304–0.683)	<0.001
Positive	Reference	
**2**–**5 years**		
Negative	0.561 (0.372–0.844)	0.006
Positive	Reference	
**Vascular invasion**		
** <2 years**		
Negative	0.536 (0.362–0.794)	0.002
Positive	Reference	
**2**–**5 years**		
Negative	0.709 (0.476–1.056)	0.091
Positive	Reference	

## Discussion

Local recurrence and distant metastasis after curative surgery in patients with CRC remain a major concern and are associated with dismal prognosis (9). Regular posttreatment surveillance of patients with CRC is conducive to identify a recurrence. Patients will benefit more from early detection and management of disease recurrence. For the heterogeneity of CRC, the relapse varies significantly with comparable clinicopathological features. Great efforts have been made to explore novel strategies which could predict early relapse by integrating clinicopathological characteristics and multigene expression patterns (10, 11). As more than 70% recurrences occurred within 2 years and over 90% occurred within 5 years after curative surgery, the frequency of follow-up gradually decreases after 5 years of curative resection. However, some patients with CRC still developed relapse after 5 years of curative surgery. It questions whether a follow-up program should last beyond 5 years to improve prognosis by identifying recurrences and metastases early when they are at a curable stage. Previous studies reported that recurrence rates were 1.2–11.6% after 5 years (12–14). No clinical guidelines are available for the effective detection of late recurrences. This study reported a higher rate of recurrence over 5 years (11.7%) than previous studies. The variations in the late recurrence rate in different studies may result from different inclusion criteria. Patients who received neoadjuvant therapy were excluded from this study. These patients were more likely to have an advanced stage of the disease and were likely to experience early relapse. Besides that, metastatic patients with CRC who are more likely to experience early relapse were not included in the study.

Age is a well-established prognostic factor in CRC (15, 16). (13) found that median ages of recurrence were higher in the late recurrence group than in the early and intermediate recurrence groups. In another study, there were no differences between the early and late recurrence groups in terms of age (8). In this study, older patients were associated with an increased risk of late recurrence. Elderly patients tend to have more indolent cells. It is hard to detect relapse in the early period because the lesions grow slowly.

Evidence from the Surveillance, Epidemiology, and End Results Program database indicated that features and survival between colon and rectal cancer were different (17). (8) found that a higher proportion of late recurrence was observed in rectal cancer as compared with colon cancer, although the difference was not statistically significant. The results were consistent with their study. For the histological grade, tumors that recurred after 5 years were more likely to be well-differentiated. It seems to be consistent with the findings of previous studies (6, 8). Indeed, patients with poorly differentiated tumors were associated with shorter disease-free survival (DFS). Patients in the late recurrence group were less likely to have signet ring cell carcinoma, which indicated a worse prognosis.

Pathological staging is the most important prognostic factor in CRC. As recurrence time is prolonged, the proportion of T3 and stage III patients are on a remarkable decline. Patients without lymph node involvement experienced more later recurrence than patients with lymph node involvement. Vascular and perineural invasions are prognostic markers of tumor aggressiveness and poor outcomes in CRC (18, 19). In patients with CRC with stage IIA disease, vascular and perineural invasions are robust indicators for implementing adjuvant chemotherapy. Fewer vascular and perineural invasions were observed in patients with recurrence after 5 years. Unfortunately, there is still no clear consensus on the mechanisms underlying such late recurrence. Evidence indicated that patients with early recurrence were more likely to have adenomatous polyposis coli mutations (20). More biomarkers are urgently needed to identify early and late relapse. In general, patients with well-differentiated pathological features are associated with an increased risk of late recurrence. Tumors with clear pathological features are more sensitive to adjuvant chemotherapy and tend to make slow progress after relapse.

The main strength of this study is that it provides large population-based evidence for the characteristics of late recurrence after radical surgery in stage I–III CRC. The results contribute to predicting patients with a high risk of late recurrence and provide personalized follow-up strategies for the selected patients. Several limitations should be addressed. The “MSI” status, the recurrence site of CRC, pretreatment CEA levels, and tumor size were not recorded in detail in the FUSCC database. The recurrence patterns (local and/or distant) were not included in the study. Additionally, data regarding the course of adjuvant chemotherapy and therapeutic regimen after recurrence were unavailable as many patients returned to the local hospital for further adjuvant treatment after surgery. Generally, patients with high-risk stage II and stage III disease received adjuvant chemotherapy with CapeOX for 6 months. In patients who had low rectal cancer with lymph node metastasis, adjuvant radiotherapy will be advised. Patients received chemotherapy with FOLFIRI after recurrence.

In conclusion, the late recurrence of CRC was associated with certain specific clinicopathological features. After 5 years of follow-up, an enhanced follow-up is still needed for the selected patients with a high risk of late recurrence.

## Data Availability Statement

The raw data supporting the conclusions of this article will be made available by the authors, without undue reservation.

## Ethics Statement

The studies involving human participants were reviewed and approved by the Ethical Committee and Institutional Review Board of the Fudan University Shanghai Cancer Center. The patients/participants provided their written informed consent to participate in this study.

## Author Contributions

XL and QL conceived this study. DL, YY, and QL improved the study design and contributed to the interpretation of results. YY and ZS collected the data. SC performed data processing and statistical analysis. DL and YY wrote the manuscript. ZS and QL revised the manuscript. All authors approved the final version.

## Conflict of Interest

The authors declare that the research was conducted in the absence of any commercial or financial relationships that could be construed as a potential conflict of interest.

## Publisher's Note

All claims expressed in this article are solely those of the authors and do not necessarily represent those of their affiliated organizations, or those of the publisher, the editors and the reviewers. Any product that may be evaluated in this article, or claim that may be made by its manufacturer, is not guaranteed or endorsed by the publisher.
