# Supplementary material for: Clinicopathological Features of Stage I–III Colorectal Cancer Recurrence Over 5 Years After Radical Surgery Without Receiving Neoadjuvant Therapy: Evidence From a Large Sample Study
Source: Front Surg. 2021 Aug 9;8:666400. doi: 10.3389/fsurg.2021.666400 (PMC8381332; doi:10.3389/fsurg.2021.666400)
Supplement: Supplementary file 1 [file Data_Sheet_1.docx]

**Supplementary Table 1** Clinicopathological features of stage I-III rectal cancer recurrence according to postoperative time.

| **Variables** |  | **Recurrence** |  |
| --- | --- | --- | --- |
|  | <2 years (N=558) | 2-5 years (N=459) | >5 years (N=141) |
| **Age** |  |  |  |
| ≤60 | 278 (49.8%) | 181 (39.4%) | 46 (32.6%) |
| >60 | 280 (50.2%) | 278 (60.6%) | 95 (67.4%) |
| **Gender** |  |  |  |
| Male | 357 (64.0%) | 276 (60.1%) | 88 (62.4%) |
| Female | 201 (36.0%) | 183 (39.9%) | 53 (37.6%) |
| **Histologic type** |  |  |  |
| Adenocarcinoma | 474 (84.9%) | 412 (89.8%) | 123 (87.2%) |
| Mucinous | 55 (9.9%) | 40 (8.7%) | 18 (12.8%) |
| Signet ring cell | 29 (5.2%) | 7 (1.5%) | 0 (0.0%) |
| **Differentiation** |  |  |  |
| Poor | 170 (30.5%) | 104 (22.7%) | 21 (14.9%) |
| Moderate | 360 (64.5%) | 340 (74.1%) | 113 (80.0%) |
| Well | 5 (0.9%) | 6 (1.3%) | 1 (0.7%) |
| Unknown | 23 (4.1%) | 9 (2.0%) | 6 (4.3%) |
| **T stage** |  |  |  |
| T1 | 18 (3.2%) | 15 (3.3%) | 10 (7.1%) |
| T2 | 78 (14.0%) | 100 (21.8%) | 38 (27.0%) |
| T3 | 167 (29.9%) | 67 (14.6%) | 2 (1.4%) |
| T4 | 295 (52.9) | 277 (60.3%) | 91 (64.5%) |
| **N stage** |  |  |  |
| N0 | 176 (31.5%) | 192 (41.8%) | 79 (56.0%) |
| N1 | 185 (33.2%) | 147 (32.0%) | 48 (34.0%) |
| N2 | 197 (35.3%) | 120 (26.1%) | 14 (9.9%) |
| **TNM stage** |  |  |  |
| I | 59 (10.6%) | 85 (18.5%) | 35 (24.8%) |
| II | 117 (21.0%) | 107 (23.3%) | 44 (31.2%) |
| III | 382 (68.5%) | 267 (58.2%) | 62 (44.0%) |
| **Perineural invasion** |  |  |  |
| Negative | 332 (59.5%) | 325 (70.8%) | 122 (86.5%) |
| Positive | 226 (40.5%) | 134 (29.2%) | 19 (13.5%) |
| **Vascular invasion** |  |  |  |
| Negative | 321 (57.5%) | 322 (70.2%) | 124 (87.9%) |
| Positive | 237 (42.5%) | 137 (29.8%) | 17 (12.1%) |

**Supplementary Table 2** Clinicopathological features of stage I-III colon cancer recurrence according to postoperative time.

| **Variables** |  | **Recurrence** |  |
| --- | --- | --- | --- |
|  | <2 years (N=629) | 2-5 years (N=390) | >5 years (N=133) |
| **Age** |  |  |  |
| ≤60 | 266 (42.3%) | 145 (37.2%) | 37 (27.8%) |
| >60 | 363 (57.7%) | 245 (62.8%) | 96 (72.2%) |
| **Gender** |  |  |  |
| Male | 358 (56.9%) | 239 (61.3%) | 76 (57.1%) |
| Female | 271 (43.1%) | 151 (38.7%) | 57 (42.9%) |
| **Histologic type** |  |  |  |
| Adenocarcinoma | 509 (80.9%) | 309 (79.2%) | 107 (80.5%) |
| Mucinous | 94 (14.9%) | 72 (18.5%) | 24 (18.0%) |
| Signet ring cell | 26 (4.1%) | 9 (2.3%) | 2 (1.5%) |
| **Differentiation** |  |  |  |
| Poor | 241 (38.3%) | 96 (24.6%) | 26 (19.5%) |
| Moderate | 370 (58.8%) | 277 (71.0%) | 97 (72.9%) |
| Well | 1 (0.2%) | 7 (1.8%) | 4 (3.0%) |
| Unknown | 17 (2.7%) | 10 (2.6%) | 6 (4.5%) |
| **T stage** |  |  |  |
| T1 | 6 (1.0%) | 12 (3.1%) | 2 (1.5%) |
| T2 | 35 (5.6%) | 43 (11.0%) | 19 (14.3%) |
| T3 | 212 (33.7%) | 76 (19.5%) | 1 (0.8%) |
| T4 | 376 (59.8) | 259 (66.4%) | 111 (83.5%) |
| **N stage** |  |  |  |
| N0 | 192 (30.5%) | 176 (45.1%) | 75 (56.4%) |
| N1 | 210 (33.4%) | 150 (38.5%) | 39 (29.3%) |
| N2 | 227 (36.1%) | 64 (16.4%) | 19 (14.3%) |
| **TNM stage** |  |  |  |
| I | 31 (4.9%) | 40 (10.3%) | 17 (12.8%) |
| II | 161 (25.6%) | 136 (34.9%) | 58 (43.6%) |
| III | 437 (69.5%) | 214 (54.9%) | 58 (43.6%) |
| **Perineural invasion** |  |  |  |
| Negative | 391 (62.2%) | 295 (75.6%) | 117 (88.0%) |
| Positive | 238 (37.8%) | 95 (24.4%) | 16 (12.0%) |
| **Vascular invasion** |  |  |  |
| Negative | 338 (53.7%) | 278 (71.3%) | 103 (77.4%) |
| Positive | 291 (46.3%) | 112 (28.7%) | 30 (22.6%) |
